# Retreatment of hepatitis C patients with pegylated interferon combined with ribavirin in non-responders to interferon plus ribavirin. Is it different in real life?

**DOI:** 10.1186/1471-2334-10-212

**Published:** 2010-07-20

**Authors:** Fernando L Gonçales, Camila A Moma, Aline G Vigani, Adriana FCF Angerami, Eduardo SL Gonçales, Raquel Tozzo, Maria HP Pavan, Neiva SL Gonçales

**Affiliations:** 1 Grupo de Estudo das Hepatites, Disciplina de Doenças Infecciosas, Departamento de Clínica Médica, Faculdade de Ciências Médicas, Universidade Estadual de Campinas, UNICAMP, São Paulo, Brazil; 2 Centro de Hematologia e Hemoterapia, Universidade Estadual de Campinas, UNICAMP, São Paulo, Brazil

## Abstract

**Background:**

More than 50% of hepatitis C viruses (HCV)-infected patients do not respond to the classical Interferon (IFN)/Ribavirin (RBV) combination therapy. The aim of this study was to evaluate the efficacy of retreatment with Peg-Interferon alpha-2b (PEG-IFN alpha-2b) plus RBV, in patients with HCV, genotypes 1 or 3, who were non-responders to the previous standard treatment with IFN/RBV.

**Methods:**

In the period 2005-2007, a total of 238 HCV chronic patients were non-responders to previous treatment with IFN plus RBV. Of these 130 agreed to be retreated with PEG-IFN alpha-2b and participated in this evaluation (90 with genotype 1 HCV and 40 with genotype 3 HCV). Patients were retreated at assisted IFN application hubs in compliance with the country's public health system rules. They received subcutaneous PEG-IFN alpha-2b, 1.5 μg, once weekly, associated with RBV, through the oral route, with doses determined according to weight (1,000 mg if weight ≤ 75 kg and 1,250 mg if > 75 kg). Patients with genotype 1 HCV were retreated for over 48 weeks and patients with genotype 3 HCV for over 24 weeks. HCV-RNA was tested by polymerase chain reaction (PCR) at baseline, at week 12, at the end of the treatment, and 6 months thereafter. The predictiveness of week 12 in the development of a sustained virologic response (SVR) was also evaluated. Patients with negative HCV-RNA at week 12 were considered as early virologic responders (EVR).

**Results:**

EVR was observed in 25% of the patients with genotype 1 HCV and in 64% of the patients genotype 3 HCV (risk = 2.075 and p-value = 0.0414). SVR was observed in 22.2% of the patients with genotype 1 HCV and in 40% with genotype 3 HCV (intention-to-treat analysis). The positive predictive value (PPV) of the HCV-RNA testing at week 12, in order to obtain the SVR, was 65% for genotype 1 and 56% for genotype 3, and the negative predictive value (NPV) was 88% for genotype 1 and 89% for genotype 3.

**Conclusions:**

PEG-IFN alpha-2b plus weight-based ribavirin is effective in re-treating previous interferon-α plus RBV failure; 22.2% of the patients with genotype 1 HCV and 40% of patients with genotype 3 HCV achieved SVR.

## Background

Initially the treatment of chronic hepatitis C (CHC) was carried out with the combination of conventional alpha-interferon (IFN-α) plus ribavirin (RBV) over 24-48 weeks according to the genotype. Progressively, IFN-α was replaced by pegylated interferon (PEG-IFN), because this was the most efficacy regime [1-3]. The primary objective of the treatment is to achieve undetectable HCV-RNA with the polymerase chain reaction (PCR). During therapy, this assay allows testing for the early virologic response (EVR) at week 12, the end of treatment virologic response (ETVR) and the sustained virologic response (SVR) at 24 weeks after therapy suspension. In large international multicentric studies, treatment-naive patients receiving PEG-IFN plus RBV showed 54-63% of SVR when considering all viral genotypes [1-3]. The treatment-naive patients infected by genotype 1 showed lower SVR rates (42-46%), as compared with those infected by genotypes 2 and 3 (76-82%). Some authors who re-treated patients without SVR with combined treatment of IFN-α plus RBV reported that it was better to retreat relapsing patients than non-responders to the previous treatment [4-9]. Authors also observed that patients who were non-responders to monotherapy or combined therapy of IFN-α plus RBV respond better than non-responders to PEG-IFN plus RBV [5,8,10].

In Brazil, the public health system (SUS) provides free drugs for the treatment of hepatitis C virus chronic patients, in compliance with a specific Ministry of Health protocol. This protocol determines that PEG-IFN is administered weekly at reference centers called "application hubs" which are outpatient departments. Under this system it is estimated that the patients fully comply with the treatment.

The primary objective of this study was to evaluate the efficacy of retreating Brazilian patients with CHC, genotypes 1 or 3, with PEG-IFN α-2b associated with RBV at a public health system university hospital. These patients were non-responders to previous conventional treatment. The secondary objective was to evaluate the early virologic response (EVR), characterized by the negativation of HCV on week 12 and its SVR predictive value.

## Methods

This was a retrospective study carried out by the Hepatitis Study Group from the Medical Sciences School at the State University of Campinas (GEHEP). In the period 2005-2007, a total of 238 HCV non-responder patients to previous treatment with interferon plus ribavirin were considered for retreatment with PEG-IFN plus RBV. Of this total, 172 had been treated at our institution of which 152 agreed to receive retreatment with PEG-IFN plus RBV. Of these, 130 met the inclusion criteria and were retreated, in compliance with the rules created by the State of São Paulo Health Secretary and the Brazilian Ministry of Health, which provided the medication. This study was approved by our Ethical Committee. The evaluation included male and female patients, over 18, chronically infected by hepatitis C virus (HCV) genotypes 1 or 3, who were non-responders to the previous treatment with IFN-α plus RBV. Data were collected on age, sex, ethnic group, alcohol abuse and the possible infection route. Patients were considered as potentially contaminated through the parenteral route when they reported receiving transfusions, injections with non-disposable syringes or needles, or sharing materials used for manicures, acupuncture or application of tattoos given in less than optimal conditions. Patients were considered as intravenous drug users (IVDU) when reporting the use of illegal drugs or stimulants (Glucoenergan^®^) through the intravenous route, in groups, and sharing syringes or needles.

At baseline, all patients showed positive serum test for HCV-RNA by polymerase chain reaction (PCR-Cobas Amplicor^® ^HCV Test - Roche Molecular Systems Inc.). All patients were negative for HBsAg and anti-HIV.

The previous treatment for HCV was carried out with IFN-α 2a or 2b (3,000,000 UI, subcutaneous, three times a week) plus RBV (administered through oral route, twice a day, weight-based dose: 1,000 mg/day for patients under 75 kg and 1,250 mg/day for those weighing over 75 kg. In Brazil this drug is provided free by the Ministry of Health in capsules of 250 mg. All patients were treated at outpatients departments. Patients with genotype 1 had been treated for over 48 weeks and those with genotype 3 were treated for over 24 weeks. Therapy was discontinued for all patients infected with genotype 1 who had positive HCV-RNA (PCR qualitative, PCR Amplicor HCV test - Roche molecular systems) at week 24. So, only non-responder patients (positive HCV-RNA at week 24) were screened for this study. Patients relapsing or who had a breakthrough in the first treatment were excluded. We included in our sample only patients who had attended every clinical evaluation of the first treatment, who had received all the drugs from the hospital pharmacy and had done all the biochemical and molecular tests requested.

Before starting the retreatment, all patients had hemoglobin > 10 g/dL, neutrophils > 1,500 cells/mm^3^, platelet > 70,000/mm^3^, albumin > 3.5 mg/dL and INR < 1.2. The bilirubin and creatinine levels were within normal values. All patients had a liver biopsy performed no more than 18 months before the start of the study, and the diagnosis was consistent with HCV. The inflammatory activity and the fibrosis grade were evaluated by the Metavir score. Patients were considered as carriers of non significant fibrosis when classified as F0, F1 and F2. Patients with F3 and F4 were considered as significant fibrosis carriers. Patients on hemodialysis, and heavy drug and alcohol users unable to comply with the treatment, were excluded. Other patients excluded had auto-immune, degenerative, renal and hematological diseases, or had other liver metabolic diseases, or those who had hypersensitivity reaction or other contraindications to the PEG-IFN plus RBV combination.

All patients were retreated with PEG-IFN α-2b associated with RBV. PEG-IFN α-2b was administered through the subcutaneous route, with a once weekly dose of 1.5 μg. All doses were administered at reference centers called "application hubs" which are outpatient departments. The 250 mg tablets of RBV were administered through the oral route, twice a day, with the dose varying according to the weight: 1,000 mg/day if the weight was less than 75 kilos and 1,250 mg/day if the weight was over 75 kilos. The retreatment period for genotype 1 was 48 weeks, while for genotype 3 it was 24 weeks.

Clinical and biochemical evaluations were performed before starting the treatment and then monthly throughout the treatment (hemogram, AST, ALT, gama-GT, TSH and free T4 dosing) in order to evaluate adverse events, tolerance and efficacy. The AST, ALT and gamma-GT levels were expressed in quotients (qALT, qAST, qGama-GT). Thus, for example, the qALT was obtained by dividing the serum ALT value by the method's highest normal value. Those with qALT > 1 had increased ALT. In the case of patients having severe adverse events or biochemical abnormalities the dose of ribavirin or PEG-IFN α-2b was reduced. In patients with hemoglobin levels lower than 8.5 mg/dL, ribavirin was suspended and when the hemoglobin levels varied between 8.5 and 10 mg/dL, the dose was reduced to half of the initial. The reduction of PEG-IFN α-2b to two-thirds of the initial dose occurred when the platelet count was lower than 30,000/mm^3 ^or when the granulocyte count was lower than 750 cells/mm^3^.

The HCV-RNA was detected by PCR at weeks 12, 24 and 48 for patients infected by genotype 1 and at weeks 12 and 24 for patients infected by genotype 3. The EVR was tested at week 12 by qualitative HCV-RNA (PCR- Cobas Amplicor^® ^HCV Test version 2.0-Roche Molecular Systems Inc.) in 104 patients, 78 (75%) with genotype 1 and 26 (25%) with genotype 3. Patients with negative PCR on this occasion were considered as complete early virologic responders (EVR). The end-of-treatment virologic response (ETVR) was tested at week 24 (genotype 3) or week 48 (genotype 1) by the same qualitative HCV-RNA test. The SVR was evaluated by a HCV-RNA test at 24 weeks after the end of the treatment.

The patients' descriptive data analysis was presented in tables for categorical variables. In order to identify risk factors for the treatment responses, uni - and multivariate Cox regression analyses were used. The adopted significance level was 5%. The computer program SAS, System for Windows (Statistical Analysis System), and version 9.1.3 Service Pack 3, SAS Institute Inc, 2002-2003, Cary, NC, USA was used.

## Results

The evaluation included 130 patients with chronic hepatitis C who were non-responders to previous treatment with IFN plus RBV, carried out at our outpatient departments. The main demographic, epidemiological and biochemical data of the 130 patients are presented in Table 1. Most of the patients were males (72%) and white (90%), and the median age was 48. Around 33% of the patients acquired the infection through the parenteral route, 21% were IVDU and in 40% of the cases the acquisition route of HCV was unknown. Alcohol abuse was present in 17% of the studied population. Of the 130 patients, 90 (70%) were infected by genotype 1 and 40 (30%) were infected by genotype 3. Only 1 (0.8%) of the infected patients showed no fibrosis according to the Metavir score (F0); 19 (14.6%) were F1, 58 (44.6%) were F2, 38 (29.2%) were F3, and 14 (10.8%) were F4. The groups, analyzed according to the genotypes, showed similar characteristics, demonstrating that no considerable bias occurred during the data statistical analysis. A small difference, however, was observed in the presence of significant fibrosis (F3 plus F4) in genotype 3 (45% of the patients against 37.8% of genotype 1). Most patients (56%) did not need a reduction in their PEG-IFN or RBV doses.

**Table 1:** Characteristics of the patient's population according to the genotypes (N = 130)

*Characteristics*	*All*	*Genotype 1*	*Genotype 3*
	*(N = 130)*	*(N = 90)*	*(N = 40)*
Men n (%)	94 (72)	63 (70)	31 (77)
Race			
Caucasian n (%)	117 (90)	79 (87)	38 (95)
Age median, years	48	46.5	49
Alcohol abuse present, n (%)	22 (17)	15 (16)	7 (17)
Possible acquisition route n (%)			
IVDU	28 (21)	15 (16)	13 (32)
Parenteral	44 (33)	33 (36)	11 (27)
Parenteral and IVDU	5 (4)	3 (3)	2 (5)
Unknown	53 (40)	39 (43)	14 (35)
Leukocytes (× 10^4 ^cells/L)	6.35	6.35	5.94
median [interval]	[2.7; 19]	[2.7; 19]	[3.9; 13]
Neutrophilis (× 10^6 ^cells/L)	3.38	3.28	5.94
median [interval]	[0.33; 9.44]	[0.33; 7.8]	[0.8; 9.44]
Hemoglobin (mg/dL)	15.3	15.5	13.5
median [interval]	[2.7; 19]	[2.7; 19]	[3.9; 13]
Platelets (× 10^3^/mm^3^)	194	184	200
median [interval]	[80; 345]	[83; 345]	[80; 275]
q ALT*	1.85	1.85	2.1
median [interval]	[0.7; 9.3]	[0.7; 5.1]	[0.9; 9.3]

qALT* = U/NUL

**Figure 1:**
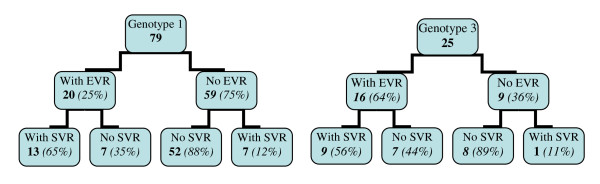
**Early virologic response (EVR) and sustained virologic response (SVR) in patients retreated with PEG-IFN alpha 2b plus RBV according to the HCV genotypes (n = 104)**.

Eleven out of 130 (8.5%) patients did not conclude the treatment due to side effects resulting from the drugs administered. These 11 patients were infected by HCV genotype 1.Of the 90 patients with genotype 1, 79 (87.7%) performed HCV-RNA testing (PCR) at week 12, while 25 (63%) of 40 patients infected by genotype 3 were also tested in the same week. All of them completed the treatment period and were tested for the presence of HCV-RNA six months after the end of the therapy to estimate the SVR rates. A higher percentage of patients with genotype 3 reached EVR (64%), when compared to genotype 1 (25%) (Figure 1).There were no significant differences in the EVR of patients infected by genotypes 1 or 3, when they were analyzed according to sex, race, mean age, alcohol abuse, and the kind of exposure or enzyme alterations of AST, ALT and gamma-GT (Table 2). In both patient groups there were no statistical differences in regard to higher or lower EVR percentages associated with the liver fibrosis grade.

**Table 2:** Early virologic (EVR) and sustained virologic response (SRV) according to the genotype and population characteristics.

*Characteristics*	*EVR Genotype 1*	*EVR Genotype 3*	*P value*	*SVR Genotype 1*	*SVR Genotype 3*	*P value*
Sex						
Men	23% (13/5)	65% (13/20)	0.9917	27% (17/62)	32% (10/3)	0.7612
Women	30% (7/23)	83% (5/6)		22% (6/27)	33% (3/9)	
Race Caucasian	28% (19/70)	60% (14/23)	0.7502	29% (14/47)	31% (12/38)	0.3662
Age						
< 40 years	33% (3/9)	100% (4/4)	0.4856	40% (4/10)	50% (3/6)	0.2694
> 40 years	28% (19/67)	59% (13/22)		24% (18/75)	29% (10/34)	
Alcohol abuse						
Yes	22% (5/22)	20% (1/5)	0.1798	29% (8/27)	12% (1/8)	1.1
No	27% (15/55)	755 (15/20)		36% (15/41)	36% (12/33)	
Exposure n (%)						
IVDU	37% (6/16)	55% (5/9)		25% (4/16)	30% (4/13)	
Parenteral	29% (8/27)	50% (3/6)	0.7038	25% (8/32)	36% (4/11)	1.073
Parenteral/IVDU	0% (0/3)	0% (0/1)	0.6794	0% (0/3)	25% (1/4)	1.053
Unknown	18% (6/32)	88% (8/9)		25% (4/16)	38% (5/13)	

p value < 0.005 = significant

By intention-to-treat (ITT) analysis the SVR was lower in patients with genotype 1(20/90,22.2%) when compared to 40% (16/40) of SVR in patients infected by genotype 3. By per protocol analysis (PPA) the SVR also was lower in patients infected by genotype 3 (20/79, 25.3%) compared to 40% (16/40) of SVR in patients infected by genotype 3. Figure 1 shows the results of the HCV-RNA testing at week 12 (EVR) in the 104 patients who performed this test and at 24 weeks after the end of the treatment. There was a statistically significant correlation for both genotypes concerning the absence of EVR and the absence of SVR (NPV = Negative Predictive Value). The positive predictive value (PPV) was 65% (13/20) for genotype 1 and 56% (9/16) for genotype 3 and the negative predictive value (NPV) was 88% (52/59) and 89% (8/9), respectively. It is important to note that 7 patients with genotype 1 and only 1 with genotype 3 reached SVR, despite presenting no EVR. In an unvaried Cox regression analysis, the sole risk factor for not obtaining EVR was the presence of infection by the genotype 1, with a risk of 2.075 (CI 95% [1.029; 4.183]) and p value below 5% (0.0414). Regarding the reduction of medication dosage, there was no statistically significant change in the SVR, with the reduction of peg-interferon or ribavirin or both. Additionally, no relation was found between the fibrosis grade and lower response to the treatment. The body mass index (BMI) was also related to EVR and SVR for each genotype, with no statistical differences between the groups.

We observed that all patients with significant fibrosis and infection by genotype 1 who had drug dose reduction did not achieve sustained virologic response, in contrast to those infected by genotype 3. When analyzing patients with non-significant fibrosis (F0, F1 and F2) who had reduced drug doses, no difference was observed in the sustained virologic response between genotypes 1 and 3. Patients with genotype 3 and non-significant fibrosis had a worse response to dose reduction than those with F3 and F4 stages.

## Discussion

Overall, our population of non-responders is similar to those of other studies and to the standard population of patients infected by HCV in our country. There was homogeneity regarding demographic, epidemiologic and biochemical characteristics among patients infected by genotypes 1 and 3, except for those who had significant fibrosis (grades 3 and 4 on the Metavir fibrosis score), whose percentage was proportionally higher for genotype 3 than genotype 1 (45% versus 37,8%).This was also seen in other Brazilian studies [4,7]. We observed a higher percentage of IVDU among those infected by genotype 3 (32%) and a higher percentage of cirrhosis carriers (20%), which could reduce the SVR rates.

It is known that patients who have relapses after the standard IFN treatment, whether combined or not with RBV, respond better to the retreatment with PEG-IFN plus RBV than those not responding to the same regimes. Krawitt et al. observed 55% of SVR in 66 relapsing patients when retreated with PEG-IFN alpha 2b (100-150 μg/week) plus RBV (1,000 mg/day) against 20% of SVR in 116 previous non-responder patients treated with the same regime [6]. They also observed SVR in 53% of the relapsing patients infected by genotype 1 and in 59% of the relapsing patients infected by genotypes 2/3. Therefore, there was no significant difference in this group of patients. This was not observed among the previous non-responders when retreated. Of these, only 17% of patients infected by genotype 1 presented SVR, as compared to 57% of the infected by the genotypes 2/3. Therefore, genotype influenced the SVR in previous non-responders. Multicentric studies, sponsored by pharmaceutical companies and conducted in Brazil by Parise et al. with PEG-IFN alpha-2a plus RBV [4] and by Gonçales Jr et al., with PEG-IFN alpha-2b plus RBV [7], in patients who were non-responders to IFN/RBV, found higher SVR percentages (24-38%) when compared to the international studies. Sherman et al. found a SVR percentage of 23% among the non-responders against a SVR of 41% among relapsing patients after retreatment with PEG-IFN alpha-2a and ribavirin [9]. In the present study, with real life patients treated outside clinical trials, we found by intention-to-treat (ITT) analysis 22.2% (20/90) of SVR in the patients infected by genotype 1 and 40% of SVR among the infected by genotype 3. By per protocol analysis the SVR also was lower in patients infected by genotype 3 (20/79, 25.3%) compared to 40% (16/40) of SVR in patients infected by genotype 3. Because patients in this study received all injections of PEG-IFN at a specialized center we should expect better rates of SVR than those treated in their homes. Again, the Brazilian patients who were non-responders to previous treatment with IFN plus RBV, when retreated, obtained good SVR rates, particularly those infected by the HCV genotype 1. The lower SVR rate observed in our Brazilian patients, infected by genotype 3, when compared to that observed by Krawitt et al. (57%) may be associated with the type of patient included by us, as 45% of them showed significant fibrosis. Additionally, Krawitt et al., in contrast to the present study, did not include non-responders to the IFN monotherapy in their retreatment group.

Two large international studies, HALT-C and EPIC3, that retreated with PEG-IFN α-2a/α-2b plus RBV HCV patients who were non-responders or relapsers to previous treatment with interferon plus RBV, obtained 18% of SVR for non-responders [5,8] and 43% of SVR for relapsers [8]. The retreatment results for EPIC3 were better in relapsers than in non-responders and, mainly, in those who received IFN plus RBV previously as compared to those who received PEG-IFN plus RBV. In EPIC3, the early virologic response (week 12) was an important predictor of SVR, as 56% of the patients with undetectable HCV-RNA obtained SVR, while no individual with decrease ≤ 2log_10 _in the serum HCV-RNA had SVR. Patients who had a decrease of at least 2 log_10 _in the viral load obtained SVR of 12%. In our study, 65% of the patients with HCV-genotype 1, with EVR, had SVR. Of the patients infected by HCV-genotype 3, 56% had SVR. It is important to note the poor liver profile of these patients (45% presented F3/F4). The negative predictive value was 88-89% showing the usefulness of carrying out the HCV-RNA testing at week 12 in the retreatment cases. With regard to week 4, there are no clear predictiveness rules for patients on retreatment. Regarding the dose reduction of medications throughout the treatment, variations were observed in the SVR obtained for each genotype. The lower SVR percentage (17%) was found in the patients who had their PEG-IFN doses reduced. It is important to point out, however, that the total sample encompassed only 17 patients who had this medication reduced. In the multivariate analysis, there was no statistically significant difference in the SVR observed among patients with or without the reduction of medications, regardless of whether ribavirin or PEG-IFN was reduced. It is probable that if the sample were more powerful it would be possible to obtain a more reliable value for the outcome of reducing pegylated interferon doses during the treatment. Recent studies have shown that the fibrosis grade is one of the primary predictors of worse therapeutic response [5,8]. When comparing the liver fibrosis grade with the SVR rate we did not find statistically significant differences between the groups of patients. In the initial stages of liver fibrosis, as evaluated by the METAVIR score (F0, F1, F2), 24% of the patients with genotype 1 obtained SVR against 25% with genotype 3, which was not significant. In advanced grades of fibrosis (F3, F4), 30% of SVR was observed in the group infected by HCV-genotype 1 against 35% in the group infected by HCV-genotype 3. In fact, in our study, the fibrosis grade itself did not appear to affect the SVR. Perhaps this may be due to a relatively small sample size of advanced fibrotics enrolled. However, when analyzing the reduction of doses of drugs, it was observed that none of the patients infected with HCV genotype 1 who had significant fibrosis and reduction of drug had a sustained virological response. In EPIC3, SVR predictors included: infection by genotypes 2/3, presence of fibrosis F2/F3, viral load ≤ 600,000 UI/ml, previous treatment with IFN monotherapy and patients relapsing after the first treatment [8]. The results of our study confirmed that the response percentage was good for Brazilian patients infected by HCV who were non-responders to the previous treatment with IFN plus RBV when retreated in real life with PEG-IFN plus RBV.

## Conclusions

Our patients, retreated at assisted interferon application hubs, had good virologic response rates. In the present study, intention-to-treat (ITT) analysis showed an SVR of 22.2% (20/90) in those patients infected by genotype 1 and 40% (16/40) among those infected by genotype 3.By per-protocol-analysis the SVR was also lower in patients infected by genotype 1 (20/79, 25.3%) compared to 40% (16/40) of SVR in patients infected by genotype 3.

## Competing interests

The authors declare that they have no competing interests.

## Authors' contributions

FLGJ participated in the design of the study, performed the statistical analysis, conceived the study, and made substantial contributions to acquisition, analysis, and interpretation. CAM, AGV, ESLG, MHP made substantial contributions to acquisition, analysis, and interpretation of data. AF performed liver biopsies and participated in the design of the study. NSLG coordinated the laboratory analyses carried out the molecular genetic studies and drafted the manuscript. All authors read and approved the final manuscript.

## Pre-publication history

The pre-publication history for this paper can be accessed here:

http://www.biomedcentral.com/1471-2334/10/212/prepub
